# Psychological Factors Influencing Appropriate Reliance on AI-enabled Clinical Decision Support Systems: Experimental Web-Based Study Among Dermatologists

**DOI:** 10.2196/58660

**Published:** 2025-04-04

**Authors:** Alisa Küper, Georg Christian Lodde, Elisabeth Livingstone, Dirk Schadendorf, Nicole Krämer

**Affiliations:** 1 Social Psychology: Media and Communication University of Duisburg-Essen Duisburg Germany; 2 Department of Dermatology University Hospital Essen Essen Germany

**Keywords:** AI reliance, psychological factors, clinical decision support systems, medical decision-making, artificial intelligence, AI

## Abstract

**Background:**

Artificial intelligence (AI)–enabled decision support systems are critical tools in medical practice; however, their reliability is not absolute, necessitating human oversight for final decision-making. Human reliance on such systems can vary, influenced by factors such as individual psychological factors and physician experience.

**Objective:**

This study aimed to explore the psychological factors influencing subjective trust and reliance on medical AI’s advice, specifically examining relative AI reliance and relative self-reliance to assess the appropriateness of reliance.

**Methods:**

A survey was conducted with 223 dermatologists, which included lesion image classification tasks and validated questionnaires assessing subjective trust, propensity to trust technology, affinity for technology interaction, control beliefs, need for cognition, as well as queries on medical experience and decision confidence.

**Results:**

A 2-tailed *t* test revealed that participants’ accuracy improved significantly with AI support (*t*_222_=−3.3; *P*<.001; Cohen *d*=4.5), but only by an average of 1% (1/100). Reliance on AI was stronger for correct advice than for incorrect advice (*t*_222_=4.2; *P*<.001; Cohen *d*=0.1). Notably, participants demonstrated a mean relative AI reliance of 10.04% (139/1384) and a relative self-reliance of 85.6% (487/569), indicating a high level of self-reliance but a low level of AI reliance. Propensity to trust technology influenced AI reliance, mediated by trust (indirect effect=0.024, 95% CI 0.008-0.042; *P*<.001), and medical experience negatively predicted AI reliance (indirect effect=–0.001, 95% CI –0.002 to −0.001; *P*<.001).

**Conclusions:**

The findings highlight the need to design AI support systems in a way that assists less experienced users with a high propensity to trust technology to identify potential AI errors, while encouraging experienced physicians to actively engage with system recommendations and potentially reassess initial decisions.

## Introduction

### Background

Even when physicians are supported by artificial intelligence (AI)–enabled clinical decision support systems (CDSSs), they are the ones who must make the final decision for ethical and legal reasons [1]. While the system only provides a suggestion, the user must decide whether to incorporate or reject the advice. Issues such as amplification of biases present in the training data, failure to generalize effectively beyond specific populations, or errors in classification can affect reliability [2-7]. Research indicates that users often struggle to find the right balance, either overtrusting and overly relying on AI recommendations or undertrusting and disregarding helpful advice [8,9]. A balance of trust is needed to appropriately rely on these systems and achieve beneficial human-AI collaboration.

As users of CDSSs are physicians with individual capabilities and levels of experience, as well as different preferences and decision-making styles, multiple factors can influence their trust in the system, their behavioral reliance on its recommendations, and whether this reliance aligns with the accuracy of the AI’s advice. Trust reflects a physician’s belief in the system’s reliability, reliance refers to the actual use of AI recommendations, and appropriate reliance describes the alignment of this use with the AI’s accuracy. Previous studies have acknowledged the importance of human factors in the interaction between physicians and clinical support systems [10-12]. However, the meta-study by Knop et al [11] does not sufficiently emphasize the importance of fostering appropriate reliance on these systems, despite listing several characteristics associated with system adoption and interaction. The characteristics identified provide a useful basis for further research into factors influencing physicians’ interactions with CDSSs. Additional research is needed to explore a broader range of factors, including trust in technology, medical experience, technology expertise, and cognitive aspects, such as confidence and the need for cognition. Together, these factors contribute to understanding how physicians interact with and appropriately rely on AI systems in their decision-making processes.

To provide a coherent framework, these factors can be grouped into cognitive aspects of prior experience and affective dimensions, such as trust. Cognitive aspects include domain-specific knowledge, such as medical experience, familiarity with and control beliefs about technology, representing technology expertise, and a willingness to engage and apply cognitive skills, measured by the need for cognition. The propensity to trust technology complements these by reflecting a baseline level of trust that users have in new systems, making it a foundational factor for reported trust and behavioral reliance on system advice [13,14]. In contrast, medical experience is treated as an additional factor that influences how users evaluate the recommendations provided by CDSS and estimate their accuracy. As clinicians gain experience, they develop a deeper understanding of clinical contexts and decision-making processes, which may influence both trust in and reliance on CDSSs [8,15-17]. Technology expertise, operationalized in this study as an affinity for technology interaction and control beliefs in interacting with technology, can influence trust and mistrust in a system, resulting in different reliance behaviors [18-20]. In addition, the need for cognition represents an individual’s enjoyment of engaging in effortful cognitive tasks and has previously been associated with different styles of advice use [21,22].

In addition to these psychological factors and medical experience, confidence in the initial decision is a key factor influencing decision-making processes in high-stakes environments, such as medical diagnosis [23-25]. Confidence in the initial decision reflects an individual’s perception of the correctness of their judgment. This can directly shape their susceptibility to external advice, including AI recommendations [26,27]. By investigating these factors, this study provides insights into the interplay between cognitive and affective dimensions that influence trust and reliance. These findings can inform the development of systems that are designed not only for acceptance, but also for effective and appropriate use, thus promoting beneficial human-AI collaboration [10,12].

Küper and Krämer [28] previously investigated these factors in a classification task based on art images and their corresponding art periods. However, their study only examined a noncritical decision-making situation performed by participants who may have had no prior experience in the decision domain. Further research is needed, particularly in critical decision-making fields, such as medicine, to ensure the generalizability of these findings to other domains. This work emphasizes expert decision-making by considering medical experience alongside psychological factors to investigate the motivation behind appropriate self-reliance and reliance on AI.

A quantitative web-based study was conducted with 223 professional dermatologists with an average of 15 (SD 11.2; range 0-50) years of experience. The study aimed to answer the research question of how the propensity to trust technology, medical experience, technology expertise, and need for cognition influence the appropriate reliance of medical personnel on CDSSs.

### Theoretical Background and Hypothesis Development

#### Overview

In the following section, we provide an overview of CDSSs and their recent achievements based on AI. The focus is on convolutional neural networks (CNNs) because the experimental setup of this study is based on image classification to simulate a realistic support situation. Furthermore, trust and reliance are differentiated and defined to clarify the terminology. Finally, the links to psychological factors and medical experience are introduced.

#### Clinical Decision Support Systems

CDSSs are valuable tools that assist clinicians in their decision-making processes by providing targeted clinical knowledge, patient information, and other relevant health data. CDSSs play a central role in managing large amounts of data [29] and ensuring the retrieval of relevant information. CDSSs do not make decisions autonomously. However, the integration of machine learning and AI, such as CNNs, has significantly enhanced their capabilities [30]. 

Among various AI systems, CNNs have been extensively used for automated image recognition, particularly in the diagnosis of skin cancer and melanoma [31]. Extensive research has shown that the accuracy of CNNs is comparable to that of dermatologists [32-34]. However, it is important to avoid portraying these systems and physicians as adversaries, as this oversimplifies the reality of CDSS. Instead, the overarching goal should be to foster a collaborative team dynamic involving a combination of humans and CNNs. As highlighted by Said et al [35], AI is designed to assist clinicians in the management and assessment of patients, and not to replace them. This collaborative approach underscores the importance of human-in-the-loop decision-making, where hybrid intelligence leverages the complementary capabilities of AI systems and human expertise to achieve superior clinical outcomes.

AI systems excel at processing large datasets, identifying subtle patterns, and providing consistent and rapid analysis, which is particularly valuable for diagnostic support [34]. Meanwhile, clinicians contribute their nuanced understanding of patient history, context, and ethical considerations to ensure that decisions align with individual patient needs [36,37]. In collaborative human-AI decision-making, dermatologists actively engage with AI systems, combining their clinical expertise with advanced technologies. The physician plays a critical role as the ultimate decision maker, determining when to trust and accept AI’s advice and when to rely on their professional judgment to ensure optimal accuracy of medical diagnoses. This approach highlights the complementary strengths of human expertise and AI capabilities, and how their integration can address each other’s limitations and improve diagnostic outcomes beyond what either could achieve independently [38].

#### Trust, Reliance, and Appropriate Reliance

In order to obtain a complete overview of the factors influencing users’ interaction with decision support systems, it is necessary to differentiate and define the different concepts discussed in previous literature. Research ranges from system acceptance and adoption [39,40] to specific investigations of trust in systems [41-44] to the calibration of trust and the appropriateness of reliance [8,9,45-49]. In the following section, we provide a brief definition of these concepts and explain which factors are important for this research.

Lee and See [13] defined trust as “the attitude that an agent will help achieve an individual’s goals in a situation characterized by uncertainty and vulnerability.” Correspondingly, reliance is the person’s behavior that is derived from trust [46]. High trust leads to reliance, while a lack of trust in a system leads to system rejection [13]. Furthermore, while too much trust can lead to overreliance, too little trust can lead to the rejection of system support. Thus, it is important to correctly calibrate trust and thereby adjust expectations about the trustworthiness of systems [26,50,51]. Correctly calibrated trust should lead to appropriate reliance, which is achieved through the human ability to distinguish between correct and incorrect AI advice and to act on this distinction [9].

The human decision maker must be able to decide when to accept and when to reject the system’s advice to achieve the highest possible decision accuracy together with the AI in a collaborative team [9]. Schemmer et al [9] further distinguish between relative AI reliance (RAIR) and relative self-reliance (RSR), which represents the percentage of cases in which the human correctly decides to rely on the AI or themselves.

We formulated the following hypothesis to test whether participants were able to distinguish between correct and incorrect AI’s advice and act on this differentiation to achieve the appropriate reliance required for optimal human-AI collaboration.

- Hypothesis 1: there is a difference in reliance on the system between receiving correct and incorrect AI’s advice. 

Chiou and Lee [46] found that trust attitudes predict reliance behavior. Previous research showed that high trust leads to blindly following the advice received [1]. We examined the influence of trust on the strength of AI’s advice use, but also looked into the appropriateness of reliance by testing for a positive influence of trust in the system on RAIR and a negative influence on RSR.

- Hypothesis 2: self-reported trust in the system has a positive influence on reliance on the system. 

An important factor for advice use is the individual’s confidence in their own decision. Sniezek and Van Swol [52] define confidence as the belief “that a specific statement, opinion, or decision is the best possible.” When humans are confident in their own decisions, they are less likely to change it after receiving advice [23,24]. We proposed the following hypothesis, assuming that participants trust advice and incorporate it into their final decision when they are less confident in their initial decision. 

- Hypothesis 3: the influence of self-reported trust in the system and reliance on the system is moderated by confidence in the initial decision. 

#### Psychological Factors and Medical Experience

##### Overview

The next section introduces psychological factors that are related to system acceptance and may therefore have an impact on trust, reliance, and thus the appropriateness of reliance, as well as the potential role of medical experience. As trust plays a central role in reliance behavior, we place it in a mediating role that influences the effect of different human factors on reliance. This is based on previous research that identified trust in the system as a mediating factor [53] that mediates the influence of personal differences on system adoption [54]. 

##### Propensity to Trust Technology

Unlike the previous concepts, the propensity to trust technology is not a state, but instead a fixed trait of trust behavior. The propensity to trust is defined as the tendency to trust others [55] and has previously been positively associated with self-reported trust [56]. Lee and See [13] link high levels of propensity to trust to a better understanding of situations in which automation advice should or should not be trusted. The following hypotheses proposed that the propensity to trust is an influencing factor for reliance on the AI system’s advice, additionally mediated by the user’s trust in the system. 

- Hypothesis 4a: the propensity to trust technology has a positive influence on self-reported trust in the system. 
- Hypothesis 4b: the propensity to trust technology has a positive influence on reliance on the system. 
- Hypothesis 4c: the influence of the propensity to trust technology on reliance on the system is mediated by self-reported trust in the system. 

##### Medical Experience

Medical experience is widely recognized as a critical factor influencing the collaborative performance of human-AI teams in medical contexts [10,18,23,57]. Knop et al [11] suggest that greater medical experience may be associated with reduced trust in AI systems, possibly due to the perception that these systems are less accurate than experienced professionals. Supporting this notion, studies have shown that individuals with more medical experience are often less susceptible to automation advice [58], whereas those with less experience are more prone to automation bias, leading to overreliance on AI [23]. However, Tschandl et al [23] found that even experienced clinicians can be misled by incorrect AI’s advice, emphasizing the nuanced nature of this relationship. Furthermore, a meta-analysis by Krakowski et al [38] highlights that clinicians of all experience levels can benefit from AI collaboration, with the greatest benefits observed for those with limited experience. On the basis of these findings, we hypothesized that greater medical experience is associated with reduced subjective trust and reliance on AI’s advice, consistent with evidence that experienced professionals are more likely to reject automation advice [58-60].

- Hypothesis 5a: medical experience has a negative influence on self-reported trust in the system. 
- Hypothesis 5b: medical experience has a negative influence on reliance on the system. 
- Hypothesis 5c: the influence of medical experience on reliance on the system is mediated by self-reported trust in the system.

##### Technology Expertise

Technology expertise has been identified as an important factor in the acceptance of and reliance on systems. However, the operationalization varies widely, encompassing perception toward automation [18], experience with computers [60], innovativeness in IT [54], past IT experience, and ability to control a new technology [59]. Given the favorable disposition toward technology inherent in many of these factors, we intend to investigate affinity for technology interaction, defined as the inclination to actively engage in extensive technological interaction, as a fundamental personal asset for adapting to technology [61]. This serves as one of the 2 measures introduced in this study to quantify technological expertise. The importance of technological experience has been highlighted in several studies as a key element for the acceptance and reliance on systems [10,18]. Consequently, we proposed the following hypotheses:

- Hypothesis 6a: affinity for technology interaction has a positive influence on self-reported trust in the system. 
- Hypothesis 6b: affinity for technology interaction has a positive influence on the reliance on the system. 
- Hypothesis 6c: the influence of affinity for technology interaction on reliance on the system is mediated by self-reported trust in the system. 

In addition, we wanted to measure control beliefs in dealing with technology to capture the nuances of technology expertise more comprehensively. Control beliefs in dealing with technology refer to an individual’s perception of his or her mastery and control over a given technology [62]. This aspect gains importance in line with the observations by Sharan and Romano [20], who emphasize that a sense of control over technology can enhance trust by reducing participants’ anxiety when interacting with the system. Therefore, in addition to the hypotheses regarding the affinity for technology interaction, we added the following 3 hypotheses, which focus on control beliefs in dealing with technology:

- Hypothesis 7a: control beliefs in dealing with technology have a positive influence on self-reported trust in the system. 
- Hypothesis 7b: control beliefs in dealing with technology have a positive influence on the reliance on the system. 
- Hypothesis 7c: the influence of control beliefs in dealing with technology on reliance on the system is mediated by self-reported trust in the system.

##### Need for Cognition

The concept of the need for cognition has been identified in various studies as an important factor influencing the acceptance of and trust in decision support systems [10,11,21]. In this research, we aimed to explore how the need for cognition influences trust and reliance on AI’s advice. The need for cognition is a personality trait that reflects an inclination to engage in mentally taxing tasks and to derive pleasure from doing so [63,64]. Brennan et al [65] found that individuals who lean toward analytical thinking, which may be associated with a high need for cognition, exhibited a higher likelihood of changing their decisions after interacting with an algorithm. Waggoner and Kennedy [21] highlighted that individuals with a high need for cognition were more likely to rely on expert advice rather than relying solely on their own mental shortcuts or heuristics. Their research strongly linked the need for cognition with trust in algorithms [21]. On the basis of these observations, we formulated the following hypotheses:

- Hypothesis 8a: the need for cognition has a positive influence on self-reported trust in the system.
- Hypothesis 8b: the need for cognition has a positive influence on reliance on the system.
- Hypothesis 8c: the influence of the need for cognition on reliance on the system is mediated by self-reported trust in the system.

## Methods

### Ethical Considerations

This study was preregistered on the Open Science Framework [66] and approved by the ethics committee of the Department of Computer Science and Applied Cognitive Science of the Faculty of Engineering of the University of Duisburg-Essen (2305SPKA7060). Informed consent was obtained from all participants via the study landing page, and the consent form is provided in the data repository linked in the Data Availability section. All data were collected anonymously, with no identifying information recorded. Participation in the study was entirely voluntary, and no financial compensation was provided.

### Procedure and Study Design

The web-based study was conducted between June 2023 and September 2023. The experimental design was based on the skin lesion classification tasks as described in the study of Vodrahalli et al [58]. It consisted of a repeated-measure experimental design in which the correctness of the AI’s advice provided was manipulated to gain insights into the appropriateness of reliance. Recruitment of participants, who were required to have a background in dermatology for expert classification of the skin lesion images, was supported by the Department of Dermatology, University Hospital Essen, and the members of the Professional Association of German Dermatologists. Most participants were recruited via a single mass email distributed to all members of the Professional Association of German Dermatologists through their official email list. To respect recipients’ time and minimize intrusiveness, no follow-up emails were sent. The recruitment email provided a detailed study description and a participation link. The recruitment period spanned from June 26, 2023, to September 25, 2023, yielding 256 responses, of which 223 (87.1%) were included in the final analysis. For transparency, a translated version of the recruitment email template is provided in the data repository linked in the Data Availability section.

At the beginning of the study, participants were informed about the procedure, data collection, and anonymization, and informed consent was obtained. In the experimental phase of the study, participants went through a 2-step decision process. Participants were presented with 24 sets of lesion images that they had to classify as benign or malignant. To do this, participants provided ratings on a continuous sliding scale marked from 0 to 100, ranging from “definitely benign” to “definitely malign.” The first decision step consisted of an unaided preliminary decision. In the second step, participants were provided with AI guidance that was reported to be 80% (19/24) accurate and displayed on a sliding scale adjacent to the participant’s original decision. Participants were informed of the AI’s overall accuracy at the start of the experiment to establish a general understanding of AI reliability. However, to avoid introducing bias, they were not informed about the correctness of individual AI recommendations while making their decisions. After completing the task, participants were presented with a summary detailing each AI classification and its accuracy to ensure full transparency.

The AI recommendations were randomly positioned between 15% and 25% from the extremes of the continuum to reflect varying confidence levels, with different placements assigned to each participant to reduce potential bias from fixed confidence levels. This randomization ensured that the confidence displayed by the AI varied across cases and participants. In the second phase of each task, participants were given the opportunity to revise their initial decision after viewing the AI’s recommendation.

To determine decision accuracy, a tailored cut-off was applied based on previous research suggesting a tendency for uncertainty near the midpoint on similar classification tasks [28]. Answers were considered correct if they fell between 61 and 100 on the scale for malignant cases and between 1 and 40 for benign cases. Responses outside these ranges were considered incorrect, allowing a nuanced classification approach based on previous evidence.

**Figure 1:**
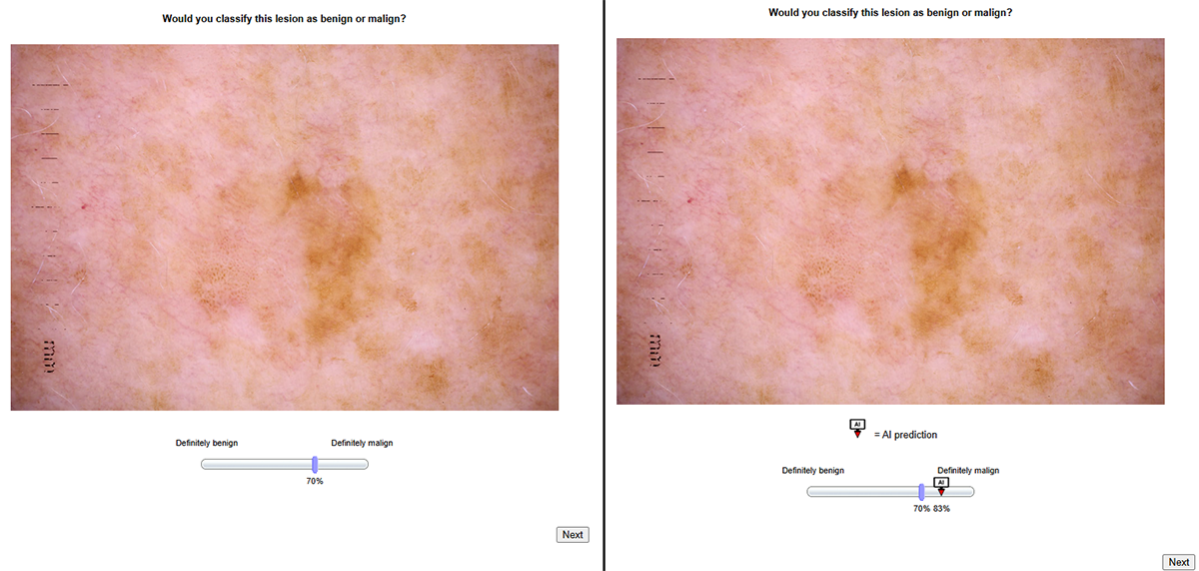
Example of the 2-step classification task.

To examine the impact of overreliance, the AI was systematically manipulated to misclassify 5 of the 24 cases presented. The misclassified cases were selected randomly, without regard to difficulty, to avoid bias in the study. To prevent order effects, the sequence of correct and incorrect AI recommendations was randomized for each participant. This randomization aimed to avoid any initial bias that might influence trust or reliance patterns based on the order of correct versus incorrect advice. Figure 1 provides a visual representation of the classification task used in the study. The figure depicts the 2-step classification task. In step 1 (left panel), participants made an initial decision by positioning the blue slider on a scale from 0% to 100%, with the slider shown here at 70%, indicating that the image was more likely to be malignant. In step 2 (right panel), the AI’s recommendation (83% malignant) was displayed, and participants could adjust the blue slider to revise their decision based on the AI’s advice.

### Dependent Variables and Confidence Measure

#### Weight of Advice

From the information collected during the experiment, which includes the first and second responses after the AI’s advice as well as the AI’s advice itself, the weight of advice (WoA) was calculated. The WoA is a commonly used metric to quantify the incorporation of advice [67] and is defined as follows:

WoA = (response_2_ – response_1_)/(advice – response_1_) **(1)**

WoA was cut off at −1 and 1 for the purpose of this study, assuming a maximum magnitude of 100%. Thus, the WoA shows a percentile change in judgment of up to 100% after receiving guidance. A positive WoA score indicates that participants have moved closer to the advice, while a negative score indicates that they have moved further away. A score closer to 1 or −1 signifies a greater shift in judgment.

#### RAIR and RSR

We wanted to go beyond the concept of WoA, and thus additionally conceptualized the appropriateness of reliance to contextualize the shift in judgment. To achieve this, we used the RAIR and RSR measures proposed by Schemmer et al [48]. These calculations were based on the accuracy of both initial and subsequent responses compared to the AI-generated advice. RAIR refers to the proportion of cases in which humans correctly adjust their initially incorrect opinions to adhere to accurate AI’s advice. Conversely, RSR refers to cases where individuals rely on their own judgment when the AI’s advice is incorrect. In both cases, a value close to 1 indicates a more appropriate level of trust, according to Schemmer et al [48].

#### Confidence

Confidence in the initial decision was also considered an important moderating factor. Rather than asking directly about confidence in the initial decision, we used the placement of a decision slider as a proxy measure. This approach follows the methodology described by Dreiseitl and Binder [27] and is supported by qualitative findings by Küper and Krämer [28], which suggests that slider positions within the outermost 20% (0-20 or 80-100) reflect high confidence, while positions in the middle range indicate varying degrees of uncertainty. Accordingly, slider positions in the ranges 0-20 or 80-100 were classified as high confidence, and values between 21 and 79 were classified as low confidence. This was operationalized as a binary measure, with 0 representing low confidence and 1 representing high confidence, to ensure consistency with previous studies and to facilitate clear interpretation.

### Questionnaires

#### Overview

In the questionnaire section of the survey, we first collected sociodemographic data, including age, gender, and years of medical experience in dermatology. We then administered the following validated questionnaires. Reliability measures were calculated on the data from this study and are reported in subsequent sections. In addition, 2 attention checks were included in the questionnaire section. These were simple multiple-choice questions designed to verify participant attention by instructing them to select specific responses. Participants failing both checks were excluded from further analysis

#### Trust

To assess trust, we used the global trust scale developed by Wischnewski et al [68]. This scale consists of 5 items that are specifically related to the AI system used previously. Participants rated their agreement on a 5-point scale, ranging from strongly agree to strongly disagree. An example item was “I believe in the system.” The internal consistency, as measured by Cronbach α, was found to be good at 0.9.

#### Propensity to Trust Technology

The propensity to trust in technology was assessed using the 6-item questionnaire developed by Jessup et al [69]. Participants indicated their level of agreement on a 5-point scale ranging from strongly disagree to strongly agree. A sample statement was “Technology is reliable.” Internal consistency, as measured by Cronbach α, was found to be acceptable at 0.8.

#### Technology Expertise

We used the Affinity for Technology Interaction scale by Franke et al [61], which consists of 9 items. Respondents expressed their agreement on a 6-point scale, ranging from completely disagree to completely agree. An example item was “I like to occupy myself in greater detail with technical systems.” Internal consistency, as assessed by Cronbach α, was found to be good at 0.8.

Control beliefs in dealing with technology were assessed using the German “Kontrollüberzeugung im Umgang mit Technik” questionnaire by Beier [62], which was translated into English for this study. This questionnaire included 8 items, with responses recorded on a 5-point scale from strongly disagree to strongly agree. For example, one of the items was “Even if I encounter resistance, I continue to work on technical problems.” The internal consistency, determined by Cronbach α, was rated as good with a value of 0.8.

#### Need for Cognition

To measure the need for cognition, we used a short 6-item scale developed by Lins de Holanda Coelho et al [64]. Items such as “I would prefer complex to simple problems” were rated on a 5-point scale, ranging from extremely uncharacteristic of me to extremely characteristic of me. The internal consistency was found to be acceptable, with a Cronbach α value of 0.8.

### Sample Size

For a regression model with 6 predictors, assuming a medium effect size (*R*^2^=0.13), a statistical power of 0.9, and a significance level of α=0.05, a sample size of n=124 would be required to detect a significant overall model [70,71]. Similarly, a 2-tailed *t* test comparing differences in the WoA between conditions of incorrect and correct advice would require n=199 datasets to detect an effect size of Cohen *d*=0.2 with a statistical power of 0.9 and a significance level of α=.05 [70,71]. On the basis of these calculations, the study aimed to include 200 participants. These sample size calculations were preregistered based on planned analyses. However, during the actual data analysis, deviations from these initial plans were made to better adapt the analysis to the structure and characteristics of the collected dataset.

A total of 256 individuals with a background in dermatology participated in the web-based study. Inclusion criteria required participants to have relevant professional expertise in dermatology. To ensure data quality, 2 attention checks were embedded in the questionnaire. Participants who failed either of these checks were excluded from the analysis, resulting in a final dataset of 223 (87.1%) participants. Of the sample, 36.3% (81/223) identified as male and 63.7% (142/223) identified as female and no one identified as nonbinary. Participants ranged in age from 26 to 78 years (mean 44.3, SD 12.2 years) and reported a mean of 15.5 (SD 11.2; range 0-50) years of experience in dermatology.

### Statistical Analysis

All quantitative analyses were performed using SPSS Statistics (version 28; IBM Corp) and SPSS Amos Graphics (version 28; IBM Corp). Means, SDs, and Pearson product-moment correlations for all variables are included in Multimedia Appendix 1.

For H1, a 2-tailed *t* test was conducted to identify differences in reliance behavior when receiving correct versus incorrect AI’s advice. A linear regression analysis was used for H2 to understand the influence of trust on reliance behavior, while a moderation analysis revealed the influence of confidence on this relationship between trust and reliance behavior (H3).

For the other hypothesis involving psychological factors and medical experience based on preregistration, a structural equation model, including propensity to trust, medical experience, affinity for technology interaction, control beliefs in interacting with technology, and need for cognition, as well as confidence in the decision and reliance behavior was calculated and estimated based on established fit criteria. The standardized root means square residual was 0.03, which was below the required 0.08, and the comparative fit index was 0.96 and thus above 0.90. The root mean square error of approximation of 0.15 was not below the required 0.08. The *χ*^2^_5_ was 12.3, which was above the required 2.00. Therefore, the statistical model was not accepted and was dropped from the analysis plan. Instead, we calculated independent mediation analyses to gain specific insights into the influence of psychological factors on subjective trust and reliance behavior.

## Results

### Accuracy and Reliance Patterns

The accuracy of each participant’s first unaided response and second response with AI support was calculated by dividing the total number of classifications (n=24) by the number of correct classifications made by the participant. There was a significant (2-tailed *t*_222_=−3.3; *P*<.001; Cohen *d*=4.5) but small difference in accuracy between the first unaided response, which had 63% (15/24; SD 8.7%) accuracy, and the final response, which had 65% (15/24; SD 9%) accuracy. On average, the accuracy increased by 1% (SD 4.5%), with a maximum gain of 17% (4/24) and a minimum decrease of 8% (2/24) after AI interaction. Differences in accuracy depending on the correctness of AI’s advice are provided in Multimedia Appendix 2, which shows an increase in accuracy of 3% (1/24) when receiving correct AI advice and a decrease in accuracy of 7% (2/24) when receiving incorrect AI advice.

Given the wide range of experience levels (0-50 years), we further examined whether experience influenced accuracy improvements with AI assistance. While overall accuracy increased by 1%, it is plausible that the less experienced dermatologists benefited more. To explore this, we conducted a subgroup analysis based on clinical expertise development, using a 5-year threshold informed by prior research [72], to distinguish between “early-career” and “established” dermatologists. The analysis showed no significant difference in accuracy improvement between the 2 groups (*t*_222_=−0.5; *P*=.32; Cohen *d*=8.7). Accuracy in dermatologists with ≤5 years of experience (62/223, 27.8%) improved from 63.51% to 64.25%, while accuracy in those with >5 years of experience (161/223, 72.2%) increased from 64.10% to 65.22%. A visualization of RSR, RAIR, and accuracy stratified by experience level is provided in Multimedia Appendix 2.

On the basis of the first and second responses in relation to the AI advice received, the interactions were classified into reliance patterns based on the study by Cabitza et al [8]. Table 1 presents the different reliance patterns, including the percentages observed in the dataset. On the basis of these, a RAIR of 10.04% (139/1384, beneficial overreliance divided by beneficial overreliance and detrimental self-reliance) and an RSR of 85.6% (487/569, beneficial self-reliance divided by beneficial self-reliance and detrimental overreliance) were calculated, showing a high level of self-reliance but a low level of AI reliance.

**Table 1:** Definition of possible reliance patterns based on the study by Cabitza et al [8] and percentages of cases^a^.

Reliance pattern	Human decision	Artificial intelligence advice	Final decision	Cases (N=5352), n (%)
Detrimental reliance	0	0	0	543 (10.15)
Beneficial underreliance	0	0	1	3 (0.06)
Detrimental self-reliance	0	1	0	1245 (23.26)
Beneficial overreliance	0	1	1	139 (2.60)
Detrimental overreliance	1	0	0	82 (1.53)
Beneficial self-reliance	1	0	1	487 (9.10)
Detrimental underreliance	1	1	0	6 (0.11)
Beneficial reliance	1	1	1	2847 (53.2)

^a^In the decision and advice columns, 0 signifies an incorrect decision point and 1 signifies a correct decision point. Detrimental signifies reliance that led to an incorrect final decision, while beneficial shows a correct final decision. In total 223 participants were presented with 24 classifications resulting in 5352 cases.

### Analysis of Appropriate Reliance, Trust, and Confidence

Hypothesis 1 postulated a difference in the strength of reliance between receiving correct and incorrect AI advice. A 2-tailed *t* test was calculated for the difference in WoA between correct and incorrect advice and showed significant results (2-tailed *t*_222_=4.2; *P*<.001; Cohen *d*=0.1). Participants relied less strongly on incorrect advice (mean change in WoA 0.1, SD 0.1) than on correct advice (mean change in WoA 0.1, SD 0.1). Thus, hypothesis 1 is supported, showing a difference in reliance behavior depending on the correctness of AI’s advice, with greater reliance on correct AI advice.

Hypothesis 2 stated that trust predicts reliance behavior. A linear regression analysis showed significant results (β=0.05; *t*_222_=3.7; *P*<.001), with trust explaining 5.8% (13/224) of the variance in reliance (*R*^2^=0.1; *F*_1,222_=13.6; *P*<.001). Further testing of RAIR and RSR showed a positive influence of trust on RAIR (β=0.05; *t*_222_=3.7; *P*<.001) explaining 24.1% (54/224) of the variance (*R*^2^=0.2; *F*_1,222_=13.9; *P*<.001), and a significant negative influence on RSR (β=0.07; *t*_222_=−3.2; *P*=.002) explaining 21% (47/224) of the variance (*R*^2^=0.2; *F*_1,222_=10.2; *P*=.002). Thus, hypothesis 2 was accepted, and trust in the system significantly predicts reliance behavior, specifically having a positive influence on RAIR and a negative influence on RSR.

Hypothesis 3 predicted that the influence of trust on reliance was moderated by the confidence of the human in their initial decision. The overall moderation model was significant, predicting 25% (56/224) of the variance (*F*_3,219_=6.3; *P*<.001). However, confidence did not moderate the influence of trust on reliance (change in *R*^2^=0%; *F*_3,219_=0.0; *P*=.88; 95% CI −0.129 to 0167). No significant moderating effects were found for RAIR (β=0.063; 95% CI −0.086 to 0.241) and RSR (β=0.049; 95% CI −0.156 to 0.272) either. Thus, hypothesis 3, which predicted that confidence would moderate the influence of trust on reliance, was not supported by our data.

### Mediations for Psychological Factors and Medical Experience

Mediation analyses for the following hypotheses were conducted using the PROCESS macro tool developed by Hayes [73], using ordinary least square regressions with unstandardized path coefficients for total, direct, and indirect effects. CIs and inferential statistics were calculated using bootstrapping with 5000 samples. Effects were considered significant if the CI did not include 0.

**Figure 2:**
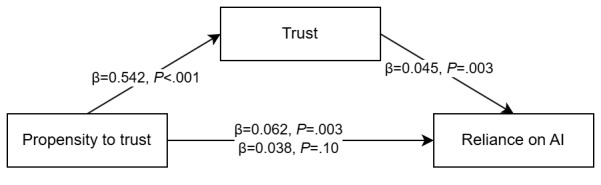
Mediation model for hypothesis 4 regarding the weight of advice. AI: artificial intelligence.

Hypotheses 4a, 4b, and 4c predicted that the influence of the propensity to trust technology on reliance on the system would be mediated by trust in the system. The beta coefficients and significance of the model paths are presented in Figure 2. The model has a significant indirect effect of 0.024 (95% CI 0.008-0.042), indicating a full mediation. In addition, there was a significant indirect effect of propensity to trust on RAIR (β=0.025; 95% CI 0.009-0.044) and a significant negative indirect effect of propensity to trust on RSR (β=−0.030; 95% CI –0.056 to –0.007). Thus, hypothesis 4, which stated that the propensity to trust technology influences reliance mediated by trust, was accepted.

**Figure 3:**
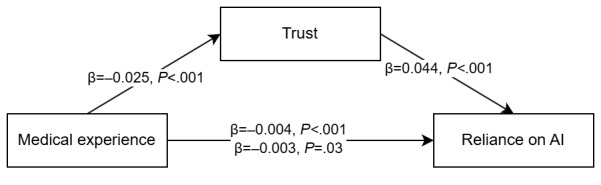
Mediation model for hypothesis 5 regarding the weight of advice. AI: artificial intelligence.

Hypothesis 5 stated that medical experience had a negative effect on trusting and relying on AI’s advice. The beta coefficients are presented in Figure 3, showing a significant partial mediation, with an indirect effect of −0.001 (95% CI −0.002 to −0.001). Furthermore, a significant negative indirect effect of medical experience on RAIR was found (β=−0.001; 95% CI −0.002 to −0.001), while there was a significant positive indirect effect for RSR (β=0.001; 95% CI 0.001-0.003). Thus, hypothesis 5, which stated that medical experience negatively predicted dependency behavior, was supported.

Hypothesis 6 postulated a positive influence of affinity for technology interaction on reliance, mediated by trust in the system. Analysis revealed no significant total effect of affinity for technology interaction on reliance (β=−0.013; *P*=.37). Affinity for technology interaction did not significantly predict self-reported trust (β=0.055; *P*=.40). In addition, no significant indirect effects were found for RAIR (β=0.003; 95% CI −0.004 to 0.010) and RSR (β=−0.004; 95% CI −0.014 to 0.005). As the direct effect of the affinity for technology interaction on reliance was not significant and affinity for technology interaction did not significantly predict self-reported trust, hypotheses 6a, 6b, and 6c were not supported. Therefore, there was no significant effect of affinity for technology interaction on trust mediated by confidence.

The influence of control beliefs in dealing with technology on reliance mediated by trust was postulated in hypothesis 7. There was no significant total effect of control beliefs on reliance (β=−0.026; *P*=.20) or on trust in the system (β=0.045; *P*=.59). In addition, there was no significant indirect effect of control beliefs on RAIR (β=0.002; 95% CI −0.006 to 0.012) and RSR (β=−0.003; 95% CI −0.016 to 0.008). Thus, hypothesis 7 was not supported, as no significant effects of control beliefs in dealing with technology on reliance or trust were found.

Hypothesis 8 proposed an influence of the need for cognition on reliance mediated by trust in the system. However, there was no significant total effect of the need for cognition on reliance (β=0.010; *P*=.60) and no significant effect of the need for cognition on trust (β=−0.062; *P*=.50). Furthermore, there were no significant indirect effects on RAIR (β=−0.003; 95% CI −0.014 to 0.006) or RSR (β=0.004; 95% CI −0.009 to 0.019). Thus, hypothesis 8, which stated that the need for cognition predicts trust and reliance, was not supported.

A comprehensive summary of the hypotheses and their respective support based on the analysis is presented in Table 2.

**Table 2:** Summary of hypothesis testing results.

Number	Hypothesis	Supported or not supported
Hypothesis 1	There is a difference in reliance on the system between receiving correct and incorrect artificial intelligence’s advice.	Supported
Hypothesis 2	Self-reported trust in the system has a positive influence on reliance on the system.	Supported
Hypothesis 3	The influence of self-reported trust in the system and reliance on the system is moderated by confidence in the initial decision.	Not supported
Hypothesis 4c	The influence of the propensity to trust technology on reliance on the system is mediated by self-reported trust in the system.	Supported
Hypothesis 5c	The influence of medical experience on reliance on the system is mediated by self-reported trust in the system.	Supported
Hypothesis 6c	The influence of affinity for technology interaction on reliance on the system is mediated by self-reported trust in the system.	Not supported
Hypothesis 7c	The influence of control beliefs in dealing with technology on reliance on the system is mediated by self-reported trust in the system.	Not supported
Hypothesis 8c	The influence of the need for cognition on reliance on the system is mediated by self-reported trust in the system.	Not supported

## Discussion

### Principal Findings

The goal of the physician’s interaction with CDSSs should be appropriate reliance—not overly relying on system advice and following incorrect recommendations or rejecting the support due to a lack of trust in the systems [8,9,45-49]. Previous research has examined factors that influence the adoption and acceptance of CDSSs [10,11,18], but a comprehensive overview of the factors that motivate appropriate reliance in AI-based medical decision support is lacking.

Given that physicians are a user group with critical expertise who are often faced with critical decision-making scenarios, it is crucial to uncover the determinants that shape the appropriate interaction between physicians and AI systems. This study aimed to investigate how psychological factors influence self-reported trust and behavioral reliance on AI systems in the medical field, specifically in the context of lesion image classification. The focus on this particular domain is crucial due to the critical nature of medical decision-making, where accurate and timely assessments are of great importance. This research examines the impact of various factors, such as propensity to trust technology, medical experience, affinity for technology interaction, control beliefs in interacting with technology, the need for cognition, and confidence, influence the appropriate reliance on medical AI. In doing so, this research not only provides essential insights but also contributes to the ethical and effective development of AI systems used in the medical decision-making process.

### Reliance, Trust, and Confidence

Most importantly, for estimating the appropriateness of reliance, a substantial difference was found between following correct and incorrect AI’s advice. Participants were less likely to follow advice when it was incorrect. In general, this study found only a very small increase in accuracy after AI interaction, which was further reflected in only 10.04% (139/1384) of RAIR, indicating a lack of reliance on AI’s advice even when the advice was correct. In contrast, the level of RSR was 85.6% (487/569). Thus, participants seemed to usually stick to their own decisions and were not influenced by the AI’s advice, even in cases where the AI’s advice was correct and theirs was not.

Comparing our results with the study by Küper and Krämer [28], in which a general audience was asked to perform art period classification tasks, our research shows notable differences. Particularly, the user group of medical professionals shows a higher degree of RSR and a lower degree of RAIR. These differences may be due to several factors, such as the expertise of medical professionals, which encourages a more critical evaluation of AI recommendations; the increased risk associated with their decisions, which reinforces the importance of cautious reliance; and, as evidenced by a lower mean subjective trust score, a general tendency to place less trust in the AI system. Essentially, these differences highlight the need to convince professionals of the value of CDSSs, with the aim of cultivating a more informed but cautious reliance on these technologies. While physicians appear to be good at maintaining their own decisions, they need additional support to be more willing to consider AI’s advice, especially in cases where AI offers a better classification compared to their own.

Analysis showed that self-reported trust significantly predicted reliance behavior. This is in line with previous research [13,28,46]. Higher levels of trust indicated greater RAIR, while lower levels resulted in more RSR. Self-reported trust explained up to 24.1% (54/224) of the variance of reliance behavior. Thus, the level of trust reported by individuals significantly influenced how much they relied on the AI, suggesting that trust plays a crucial role in shaping their interaction with the system.

Because high levels of both RAIR and RSR would lead to the most appropriate reliance behaviors, these results underscore the importance of properly calibrating trust. Proper trust calibration involves relying on AI’s advice when it is accurate (RAIR) and trusting one’s own judgment when the AI is likely to be incorrect (RSR). Achieving the highest accuracy requires this balance—not blindly following AI recommendations when they are wrong, while remaining open to its suggestions when they are likely to be correct. The ability to calibrate trust effectively ensures that both AI guidance and personal expertise are used appropriately in decision-making.

Confidence in the decision does not significantly moderate the influence of trust on reliance behavior. However, confidence is negatively correlated with the WoA and has a significant positive correlation with RSR. Thus, participants with high confidence in their initial decision were less likely to follow the AI’s advice and thus were more likely to stick with their initial decision.

Previous studies [23,24,47] have found a similar relationship between confidence and following advice. Our hypothesis that predicted a moderating role of confidence on the influence of trust on reliance behavior was not supported. However, confidence was highly correlated with medical experience and RSR, and negatively correlated with trust in the system and WoA. Thus, as predicted, high confidence in the initial decision appears to be negatively related to advice use. In addition, confidence correlated with accuracy before and after AI use, suggesting that participants were particularly confident in their decision when the decision was actually correct. Thus, this high confidence in itself would not be detrimental to beneficial human-computer interaction, as there was no significant relationship with RAIR, indicating that participants did not generally follow AI’s advice less because of high confidence, but did so in cases where they believed the AI’s advice was incorrect.

### Psychological Factors and Medical Experience

A key aim of this study was to identify human characteristics that are important in predicting trust and reliance on CDSSs.

Our results show that the propensity to trust technology is indeed an important influencing factor for trust development and reliance behavior, with a high propensity to trust leading to higher levels of trust in the specific system. In contrast, a high level of medical experience signified a skeptical attitude toward the system, resulting in lower self-reported trust and AI reliance behavior. No significant effects were found for affinity for technology interaction, control beliefs in interacting with technology, and the need for cognition.

The significant positive influence of the propensity to trust technology on reliance behavior is further strengthened by the self-reported trust in the system, which acts as a mediator. Thus, physicians with a higher propensity to trust in technology are more likely to be influenced by AI’s advice and to follow correct advice, but, as indicated by the negative influence on RSR, are also more likely to be diverted from correct decision-making when given incorrect system advice.

This extends previous research focusing on trust formation [20,24] by differentiating reliance by RAIR and RSR and highlighting the role of self-reported trust as a mediator. A high propensity to trust technology indirectly influences RAIR positively by making the participants more susceptible and acceptant of system advice, while a low propensity to trust technology is associated with high levels of self-reliance. Particularly, users with a high propensity to trust technology are at risk of mistakenly relying on and following erroneous AI’s advice and therefore need additional information to help them differentiate between good and bad system advice.

Our research identified medical experience as a significant factor influencing self-reported trust and reliance behavior, which is consistent with previous research [58-60]. Higher levels of medical experience led to lower levels of trust in and reliance on the system, with trust reinforcing this negative influence of medical experience on reliance. Knop et al [11] identified similar results, suggesting that experienced professionals believed the systems to be less accurate, leading to lower levels of trust. Medical experience also led to higher RSR, indicating that more experienced physicians were less likely to be misled by incorrect AI’s advice, contradicting previous research by Tschandl et al [23]. There was no significant correlation between years of medical experience and the accuracy of the first or second response. Given the broad range of experience levels among participants, we further explored how medical experience influenced accuracy. While it is plausible that less experienced dermatologists might benefit more from AI support, a subgroup analysis revealed no significant difference in accuracy improvement between groups. These findings suggest that experience level did not significantly moderate the effectiveness of AI support in improving diagnostic accuracy. However, the observed patterns in reliance behavior, as visualized in the Multimedia Appendix 2, underscore the need for further investigation into how expertise influences AI-assisted decision-making. Factors, such as the type of AI explanations provided, the clarity of the model’s reasoning, or even cognitive biases, such as automation bias and overreliance, could play a role in how both experienced and inexperienced physicians engage with AI assistance. Vodrahalli et al [58] identified task expertise as a barrier to accepting system advice, which was replicated in this study. While complementary expertise is the basis for collaborative human-AI decision-making [74], participants with high medical experience appear to need additional cues to build trust in the AI and make it easier to identify when it might be beneficial to follow AI suggestions. This could be achieved by including explanations for the AI recommendations, in line with the principles of explainable AI. By providing a contextual background to the AI’s decisions, explainable AI supports physicians in continually reassessing their own decisions, fostering a collaborative, human-in-the-loop dynamic that is essential for hybrid intelligence in medical decision-making [75-78]. Research has shown that explanations generally increase user trust in AI systems [79,80], promoting transparency and trust. Metta et al [77] demonstrated that explainable AI can improve trust and confidence in systems assisting with skin lesion diagnosis. Similarly, Chanda et al [79] found that while AI support increased clinicians’ trust and diagnostic accuracy compared to no AI assistance, the addition of explainable AI did not further enhance accuracy. In contrast, Tschandl et al [23] found that explanations helped teach medical students to focus on specific diagnostic factors, thereby improving their performance. Further research is needed to uncover the interplay between explainable AI and the appropriateness of reliance.

Affinity for technology interaction showed no significant influence on self-reported trust or reliance behavior. Furthermore, control beliefs in interacting with technology did not have a significant effect on self-reported trust in the system or reliance behavior. This contradicts previous research that has identified technology expertise as an important factor in system adoption and interaction [10,18]. However, it provides a positive perspective, suggesting that health care professionals look beyond their attitudes toward technology and instead focus on their own experience in the field when judging the advice of an AI system.

Finally, the physicians’ need for cognition did not have a significant influence on trust or reliance, as suggested by previous research by Brennan et al [65]. As this study presented a simplified support situation that only provided the final classification of AI and no additional explanation as to how the systems reached this conclusion, the human-AI interaction may not have been engaging enough to elicit the relevance of a high level of need for cognition. As the advice from this AI only provided a final recommendation and no additional explanation to engage with, it is possible that the importance of the need for cognition was overestimated for this classification task. Future studies should investigate how the influence of a high need for cognition might change if the systems additionally presented explanations that might motivate more cognitive engagement when considering the advice provided by the AI.

In summary, in contrast to previous studies on entertainment decisions that used convenience samples [28], this study was conducted with medical professionals who were recruited with considerable effort. Therefore, this study provides insights specific to the physician user base and shows significant differences from the results of the previous study by Küper and Krämer [28], which was conducted in a noncritical, nonexpert decision situation where participants classified art images into art periods, representing a low-risk low-involvement classification task. Küper and Krämer [28] underline the importance of factors connected to the relationship to technology, such as the propensity to trust technology, affinity for technology interaction, and control beliefs in interacting with technology. In contrast, for the specific user group of dermatologists, this study showed similar results only for the propensity to trust technology. This may be due to the different levels of expertise. In the noncritical, nonexpert classification tasks, participants may have been less knowledgeable in the field and therefore could not base their decision on their expertise and instead were more trusting toward the classification of a trained AI. In contrast, the dermatologists in this study reported a mean experience of >15 years, making them knowledgeable in the field. This is representative of the significant influence of medical experience on trust and reliance. Furthermore, it should be highlighted that the results of hypothesis 1 indicate that they were able to differentiate between correct and incorrect to a certain extent, as they were less likely, but not consistently able, to reject incorrect advice. This is in contrast to the study of the noncritical nonexpert classification, which did not target participants with the expertise to successfully make these distinctions. Thus, it can be concluded that task expertise plays an important role in determining appropriate reliance, but the relationship is bidirectional. Although physicians showed some ability to reject incorrect AI’s advice, further research is needed to understand how experienced physicians perceive and use correct AI recommendations, particularly in the context of uncertainty surrounding the advice. Given that decision accuracy on incorrect advice cases remained low and decreased further with AI assistance, future research should explore the underlying factors that challenge physicians in recognizing and rejecting incorrect AI recommendations. The significant relationship between the propensity to trust technology and medical experience highlights the importance of CDSSs in providing additional information to help physicians calibrate their trust and rely appropriately.

### Limitations and Future Research

As discussed earlier, this study only offered the final classification of the simulated AI. Therefore, participants were not able to engage with additional information, such as explanations or further background on the validity of the AI. Future studies could include these factors to investigate whether explanations can help experts look beyond their own experience and engage more with AI’s advice. As suggested in the study by Buçinca et al [22], this may also increase the relevance of high need for cognition, which was not identified as an influencing factor in this study.

Furthermore, as this study only offered a perceptual classification task without additional information about the patient and no advice explanation provided by the AI system, the cognitive engagement required by this task may have been too low to confirm the relevance of the need for cognition. Therefore, future studies should pay particular attention to explainable AI and how the addition of explanations might affect appropriate reliance based on cognitive engagement, measured beyond the need for cognition.

Confidence was assessed using a proxy measure and then dichotomized, following approaches used in previous studies. Directly asking participants to report their confidence in both initial and AI-assisted decisions may encourage greater reflection, potentially yielding a more accurate and sensitive measure of confidence than relying on decision slider positions. Future studies could also further explore the direct effects of confidence on reliance behaviors in greater depth.

Finally, AI misclassified cases were randomly selected, which may have unintentionally introduced variability in case difficulty, potentially influencing reliance and accuracy results. Future studies should carefully control for case difficulty to clarify its impact on reliance behaviors and accuracy when interacting with AI recommendations.

### Conclusions

The research question of this study was how the propensity to trust technology, medical experience, technological expertise, and need for cognition influence the trust in and reliance on AI-enabled CDSSs. Our findings extend prior research [10,11,23] by addressing the appropriateness of reliance on these systems.

The study revealed that medical professionals often prioritize their own judgments, exercising caution in adopting AI’s advice, even when correct. The propensity to trust technology and medical experience emerged as key factors influencing trust and reliance. A higher propensity to trust was associated with greater openness to AI recommendations, while medical experience was linked to greater self-reliance and measured adoption of AI’s advice.

These results underscore the need to design AI-enabled systems that complement diverse decision-making styles. For less experienced users, fostering informed reliance while highlighting system limitations is critical to prevent overreliance. For experienced professionals, systems should facilitate meaningful engagement with AI, supporting a collaborative process that enhances diagnostic accuracy and decision-making efficiency.

Further research is essential to refine the design of CDSSs that elicit appropriate trust and confidence, ensuring effective collaboration between human expertise and AI to improve clinical outcomes.

## Appendix

Multimedia Appendix 1 Correlation analysis among research variables and descriptive statistics.

Multimedia Appendix 2 Visualization of differences in accuracy depending on the correctness of artificial intelligence’s (AI’s) advice and relative self-reliance, relative AI reliance, and accuracy stratified by experience level.

## Abbreviations

AI: artificial intelligence
CDSS: clinical decision support system
CNN: convolutional neural network
RAIR: relative artificial intelligence reliance
RSR: relative self-reliance
WoA: weight of advice

## Data Availability

The datasets generated or analyzed during this study, including the questionnaires used are available in the data repository in the associated project included in the Open Science Framework link of the study by Küper [66] in the subfolder “Dataset_Derma.”
