# Supplementary material for: Psychological Factors Influencing Appropriate Reliance on AI-enabled Clinical Decision Support Systems: Experimental Web-Based Study Among Dermatologists
Source: J Med Internet Res. 2025 Apr 4;27:e58660. doi: 10.2196/58660 (PMC12008695; doi:10.2196/58660)
Supplement: Multimedia Appendix 1 [file jmir_v27i1e58660_app1.pdf]

Multimedia Appendix 1

Table 1. Correlation analysis (Pearson *r* and two-tailed *P* value) among the research variables.

Variable		Trust	Propensity to trust	Medical Experience	Affinity for technology interaction	Control beliefs in dealing with technology	Need for cognition	Confidence in initial decision	Accuracy before AI advice	Accuracy with AI advice	Weight of advice	Weight of correct advice	Weight of incorrect advice	Relative AI reliance	Relative self-reliance
**Trust**															
	r	1	0.4[b]	-0.3[b]	0.1	0	0	-0.2[b]	0.1	0.1	0.2[b]	0.2[b]	0.2[b]	0.2[b]	-0.2[b]
	P value	–[c]	<.001	<.001	.38	.58	.51	<.001	.14	.05	<.001	.001	.01	<.001	.002
**Propensity to Trust**															
	r	0.4[b]	1	-0.3[b]	0.3[b]	0.3[b]	0.1	-0.1	0	0	0.2[b]	0.2[b]	0.1	0.1[a]	-0.2[a]
	P value	<.001	–	<.001	<.001	<.001	.26	.39	.99	.94	.004	.004	.07	.04	.01
**Medical Experience**															
	r	-0.3[b]	-0.3[b]	1	-0.1	-0.2[b]	0	0.2[b]	-0.1	0	-0.2[b]	-0.2[a]	-0.2[b]	-0.1	0.2[b]
	P value	<.001	<.001	–	.36	.01	.42	.001	.31	.78	.002	.03	.002	.14	.003
**Affinity for technology interaction**															
	r	0.1	0.3[b]	-0.1	1	0.6[b]	0.4[b]	0.2[a]	0	0	-0.1	-0.1	-0.1	0	0
	P value	.38	<.001	.36	–	<.001	<.001	.03	.84	>.99	.36	.34	.14	.53	.85
**Control beliefs in dealing with technology**															
	r	0	0.3[b]	-0.2[b]	0.6[b]	1	0.4[b]	0.1	0	0	-0.1	-0.1	-0.2[a]	-0.1	0
	P value	.58	<.001	.01	<.001	–	<.001	.13	.72	.93	.17	.26	.01	.33	.72
**Need for cognition**															
	r	0	0.1	0	0.4[b]	0.4[b]	1	0.1[a]	0.1	0.1	0	0.1	0	0.1	-0.1
	P value	.51	.26	.42	<.001	<.001	–	.04	.24	.16	.63	.38	.61	.36	.23
**Confidence in initial decision**															
	r	-0.2[b]	-0.1	0.2[b]	0.2[a]	0.1	0.1[a]	1	0.3[b]	0.3[b]	-0.1[a]	-0.1	-0.1	0	0.2[b]
	P value	<.001	.39	.001	.03	.13	.04	–	<.001	<.001	.049	.08	.08	.60	.005
**Accuracy before AI advice**															
	r	0.1	0	-0.1	0	0	0.1	0.3[b]	1	0.9[b]	-0.1	-0.1	0	0	0
	P value	.14	.99	.31	.84	.72	.24	<.001	–	<.001	.24	.26	.83	.69	.48
**Accuracy with AI advice**															
	r	0.1	0	0	0	0	0.1	0.3[b]	0.9[b]	1	0	0.1	0	0.3[b]	0.2[b]

	P value	.05	.94	.78	>.99	.93	.16	<.001	<.001	–	.68	.28	.51	<.001	.01
**Weight of advice**															
	r	0.2[b]	0.2[b]	-0.2[b]	-0.1	-0.1	0	-0.1[a]	-0.1	0	1	0.9[b]	0.7[b]	0.6[b]	-0.6[b]
	P value	<.001	.004	.002	.36	.17	.63	.049	.24	.68	–	<.001	<.001	<.001	<.001
**Weight of correct advice**															
	r	0.2[b]	0.2[b]	-0.2[a]	-0.1	-0.1	0.1	-0.1	-0.1	0.1	0.9[b]	1	0.6[b]	0.7[b]	-0.5[b]
	P value	.001	.004	.03	.34	.26	.38	.08	.26	.28	<.001	–	<.001	<.001	<.001
**Weight of incorrect advice**															
	r	0.2[b]	0.1	-0.2[b]	-0.1	-0.2[a]	0	-0.1	0	0	0.7[b]	0.6[b]	1	0.4[b]	-0.7[b]
	P value	.01	.07	.002	.14	.01	.61	.08	.83	.51	<.001	<.001	–	<.001	<.001
**Relative AI reliance**															
	r	0.2[b]	0.1[a]	-0.1	0	-0.1	0.1	0	0	0.3[b]	0.6[b]	0.7[b]	0.4[b]	1	-0.3[b]
	P value	<.001	.04	.14	.53	.33	.36	.60	.69	<.001	<.001	<.001	<.001	–	<.001
**Relative self-reliance**															
	r	-0.2[b]	-0.2[a]	0.2[b]	0	0	-0.1	0.2[b]	0	0.2[b]	-0.6[b]	-0.5[b]	-0.7[b]	-0.3[b]	1
	P value	.002	.01	.003	.85	.72	.23	.005	.48	.01	<.001	<.001	<.001	<.001	–

[a]The correlation is significant at a significance level of .05 (two-tailed).

[b]The correlation is significant at a significance level of .01 (two-tailed).

[c]Not applicable.

Multimedia Appendix 2

Table 2. Descriptive statistics for the research variables (N=223).

Variable	Mean (SD)
Trust	2.9 (0.9)
Propensity to trust	3.7 (0.6)
Medical experience	15.5 (11.2)
Affinity for technology interaction	4 (0.9)
Control beliefs in dealing with technology	3.5 (0.7)
Need for cognition	3.7 (0.6)
Confidence in initial decision	0.6 (0.2)
Accuracy before AI advice	63.9 (8.7)
Accuracy with AI advice	65 (9)
Weight of advice	0.2 (0.2)
Weight of correct advice	0.1 (0.1)
Weight of incorrect advice	0.1 (0.1)
Relative AI reliance	0.1 (0.2)
Relative self-reliance	0.8 (0.3)
