# Supplementary material for: Psychological Factors Influencing Appropriate Reliance on AI-enabled Clinical Decision Support Systems: Experimental Web-Based Study Among Dermatologists
Source: J Med Internet Res. 2025 Apr 4;27:e58660. doi: 10.2196/58660 (PMC12008695; doi:10.2196/58660)

Multimedia Appendix 2

Visualization of differences in accuracy depending on the correctness of AI's advice.

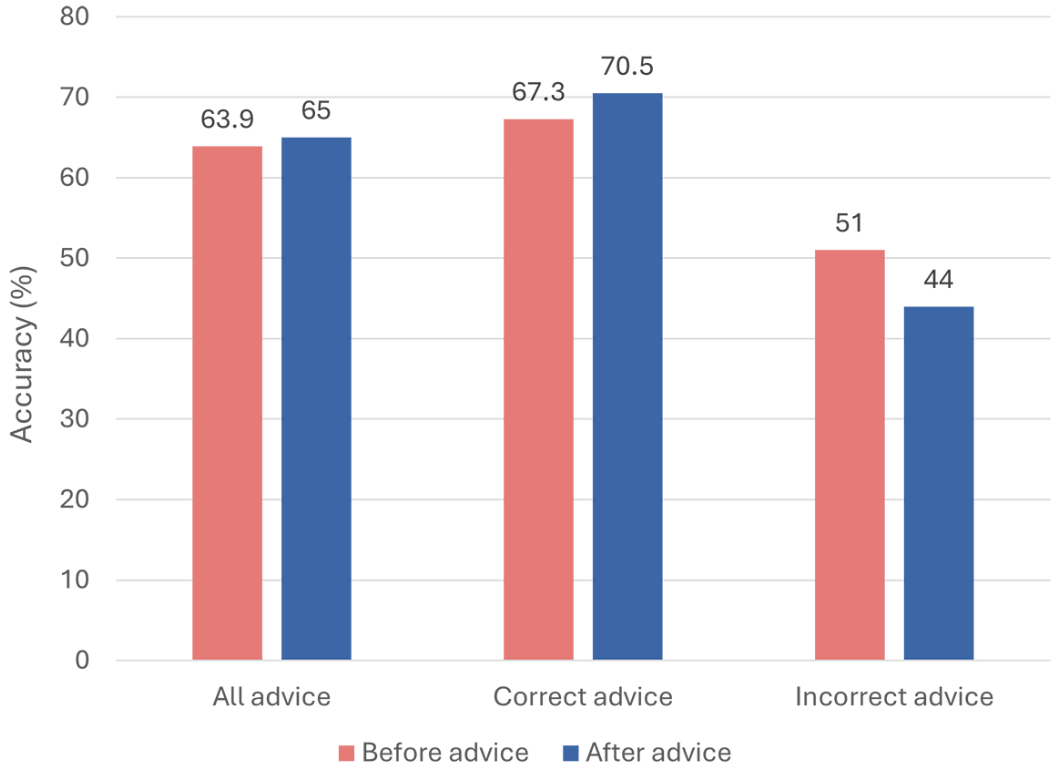

Visualization of RSR, RAIR, and accuracy stratified by experience level.

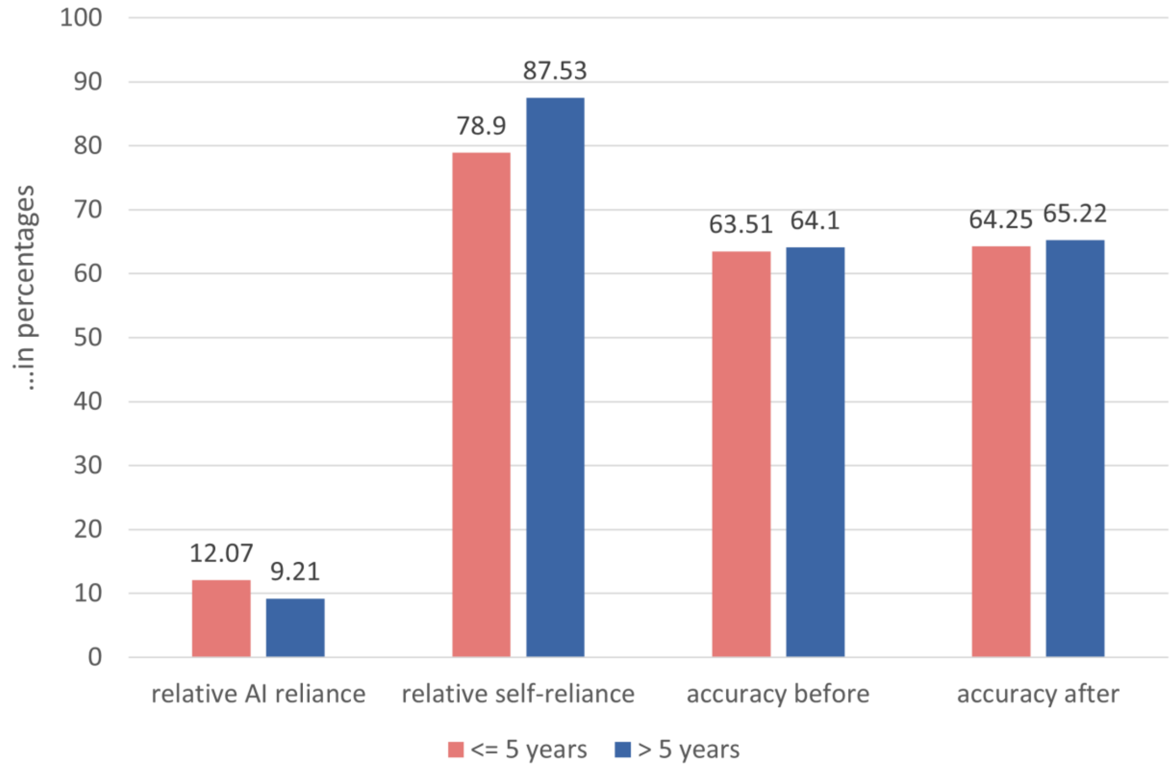

Supplement: Multimedia Appendix 2 [file jmir_v27i1e58660_app2.pdf]
